# A bibliometric and visual analysis of research trends and hotspots in sepsis-related immunosuppression (2005–2025)

**DOI:** 10.3389/fphar.2026.1755295

**Published:** 2026-04-09

**Authors:** Jiating Bao, Hongmei Gao, Jiawei Jiang

**Affiliations:** 1 Department of Intensive Care Unit, Tianjin First Central Hospital, School of Medicine, Nankai University, Tianjin, China; 2 Key Laboratory for Critical Care Medicine of the Ministry of Health, Tianjin First Central Hospital, Tianjin, China; 3 Emergency Medicine Research Institute, Tianjin First Central Hospital, Tianjin, China

**Keywords:** bibliometrics, immunosuppression, research hotspots, research trends, sepsis

## Abstract

**Background:**

Sepsis-induced immunosuppression is a major global healthcare challenge, and advancing related research is crucial for improving patient prognosis.

**Method:**

Relevant publications were sourced from the Web of Science Core Collection (WoSCC) database, with additional verification conducted through the PubMed database. The analysis and visualization of the data were executed utilizing CiteSpace and VOSviewer.

**Results:**

A total of 2633 documents were included, and the publishing volume showed a trend of “starting-rapid growth-platform period”. The countries, institutions, journals, and researchers with the largest number of documents were the United States, INSERM, *Frontiers in Immunology*, and Guillaume Monneret. The journal *Critical Care* was the most frequently cited, while Richard S. Hotchkiss emerged as the most frequently cited researcher. Research hotspots included immune cell failure post-sepsis, activation of the PD-1/PD-L1 pathway, the proliferation of myeloid-derived suppressor cells, biomarker research, and the application of immunotherapy. Furthermore, the application of immune checkpoint inhibitors and the mechanisms underlying immune cell apoptosis may be potential emerging topics in the field.

**Conclusion:**

This study provides a valuable reference for subsequent research endeavors. Future research should prioritize elucidating the molecular mechanisms underlying immunosuppression, identifying reliable biomarkers, and developing effective therapeutic strategies to improve the prognosis of patients with sepsis.

## 1 Introduction

Sepsis, characterized by life-threatening organ dysfunction resulting from a dysregulated host response to infection, continues to pose a significant challenge in global healthcare (Singer et al., 2026). In 2017, it was estimated that there were 48.9 million cases of sepsis worldwide, leading to 11 million deaths, which accounted for 19.7% of all global mortality (Rudd et al., 2020). Although age-adjusted mortality rates exhibited minimal change from 1999 to 2019, there was a marked 30.22% increase between 2019 and 2021, primarily due to the COVID-19 pandemic (Morrissey et al., 2025). Among the intricate pathophysiological mechanisms underlying sepsis, immunosuppression has been identified as a crucial determinant of adverse outcomes (Gao X. et al., 2025). This immunosuppressive state is characterized by the aberrant apoptosis of immune effector cells, excessive proliferation of immunosuppressive cells such as myeloid-derived suppressor cells (MDSCs) and regulatory T cells (Tregs), the release of anti-inflammatory cytokines, and the upregulation of immune checkpoint molecules, including PD-1/PD-L1 and Tim-3 (Zhang X. et al., 2024; S et al., 2025; Tramper et al., 2025; Gao Q. et al., 2025; Wang et al., 2025). These alterations impair pathogen clearance, increase susceptibility to secondary infections, prolong hospital stays, and elevate mortality, underscoring the urgent need to understand and target immunosuppression in sepsis management (Deinhardt-Emmer et al., 2025).

Recent advances have begun to elucidate the molecular mechanisms driving sepsis-related immunosuppression. Key pathways involve Fas/FasL-mediated lymphocyte apoptosis, metabolic reprogramming of immune cells (e.g., the shift from oxidative phosphorylation to glycolysis in macrophages), and persistent activation of inhibitory signaling pathways such as PD-1/PD-L1 (Islam et al., 2024; Yang K. et al., 2025; Gossez et al., 2025). Despite these insights, significant knowledge gaps remain regarding the dynamic interplay of these mechanisms, their temporal evolution, and their heterogeneity across patient populations. Furthermore, although immunomodulatory therapies, including immune stimulatory factors and immune checkpoint inhibitors, have demonstrated potential in preclinical and early clinical studies, their translation to routine clinical practice is hindered by the lack of robust biomarkers to guide patient selection and treatment timing (Gao Q. et al., 2025; Xiao et al., 2025; Lee et al., 2025). These unresolved questions highlight the necessity for a systematic synthesis of existing research to inform future directions.

Considering the rapidly expanding and fragmented body of literature on sepsis-related immunosuppression, traditional narrative reviews may fall short in effectively capturing the intellectual structure and evolving frontiers of this field (Chen et al., 2024). Bibliometric analysis provides a robust methodological approach to address this limitation by quantitatively mapping research trends, identifying key contributors, and visualizing emerging areas of interest. This study employs CiteSpace and VOSviewer to systematically analyze publications from 2005 to 2025, thereby delineating the global research landscape of sepsis-related immunosuppression. Our primary objectives are to: (a) characterize publication trends and key contributors (countries, institutions, journals, and authors); (b) identify research hotspots and their evolution through keyword and co-citation analysis; and (c) forecast emerging topics to guide future investigations. By providing a comprehensive and objective overview, this study aspires to serve as a valuable reference for researchers, thereby facilitating targeted efforts to elucidate underlying mechanisms, develop biomarkers, and optimize immunotherapeutic strategies, with the ultimate goal of enhancing outcomes for patients with sepsis.

## 2 Materials and methods

### 2.1 Data collection

The dataset utilized in this study is derived from the Web of Science Core Collection (WoSCC) and the PubMed database. The WoSCC database was selected as the primary data source due to its comprehensive citation data and standardized metadata, which are essential for bibliometric analysis. PubMed was employed as a validation database to assess the robustness and generalizability of findings.

#### 2.1.1 Retrieval strategy and formula

The literature retrieval was conducted with a time span of 1 January 2005, to 1 May 2025, and the retrieval strategy was based on topic terms (Title/Abstract) to ensure the relevance of included literature. The detailed retrieval formulas for each database are as follows:

WoSCC database: #1: TS = (Sepsis OR Septicemia* OR Pyemia* OR Pyaemia* OR Pyohemia*); #2: TS = (immunosuppression*); Final retrieval formula: #1 AND #2.

PubMed database: #1: ((((((((Sepsis [Title/Abstract]) OR (Septicemia [Title/Abstract])) OR (Septicemias [Title/Abstract])) OR (Pyemia [Title/Abstract])) OR (Pyemias [Title/Abstract])) OR (Pyaemia [Title/Abstract])) OR (Pyaemias [Title/Abstract])) OR (Pyohemia [Title/Abstract])) OR (Pyohemias [Title/Abstract]); #2: (immunosuppression [Title/Abstract]) OR (immunosuppressions [Title/Abstract]); Final retrieval formula: #1 AND #2.

#### 2.1.2 Inclusion and exclusion criteria

Inclusion criteria: (Singer et al., 2026): Research content is closely related to sepsis-related immunosuppression; (Rudd et al., 2020); Publication time is within the range of 2005–2025; (Morrissey et al., 2025); Language is limited to English to ensure the uniformity of literature analysis; (Gao X. et al., 2025); Publication type is restricted to original articles and reviews (the two most common and influential types in bibliometric research) to exclude conference abstracts, letters, editorials, and book chapters with incomplete research content.

Exclusion criteria: (Singer et al., 2026): Publications with irrelevant research topics (e.g., only focusing on sepsis without involving immunosuppression, or only studying immunosuppression in other diseases); (Rudd et al., 2020); Non-English publications; (Morrissey et al., 2025); Non-article/review publication types.

#### 2.1.3 Keyword consolidation

In the integration of keywords, standardized terms, unified abbreviations, or full names are preferred, specific terms are merged, and irrelevant or common terms are excluded. For example, “Septicemia*”, “Pyemia*”, “pyaemia*” and “pyohemias*” were merged into “Sepsis”; “myeloid-derived suppressor cell*,” “MDSC*,” and “myeloid derived suppressor cell*” were standardized as “MDSCs”; Delete some meaningless keywords, such as “cell*”, “line*” and “gene*”.

**FIGURE 1:**
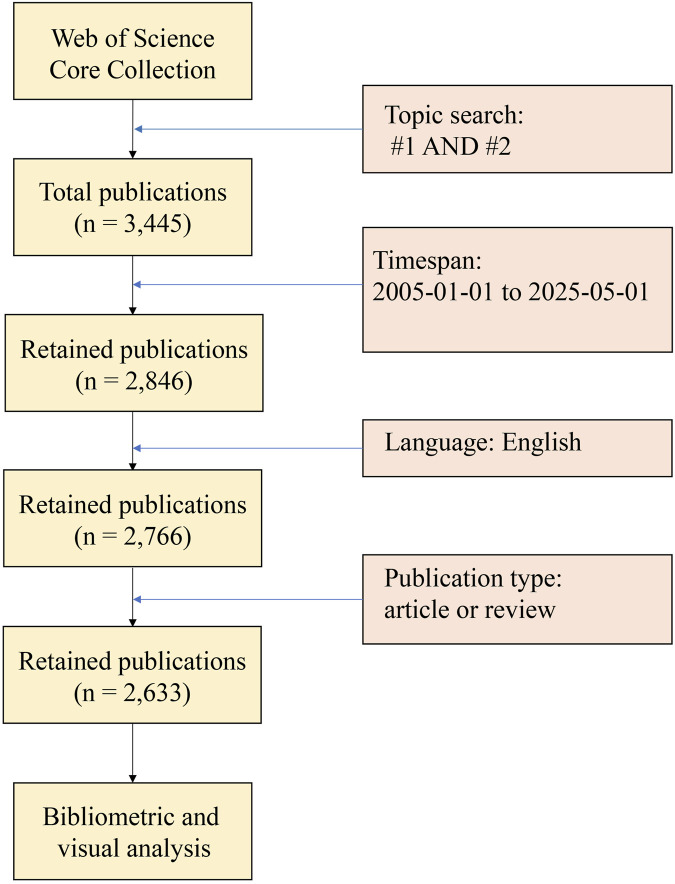
The literature search process for this study. The primary data source was the Web of Science Core Collection (WoSCC) database, with a topic search strategy of “sepsis-related terms AND immunosuppression*” and a time span of 1 January 2005, to 1 May 2025. A total of 3,445 initial publications were retrieved, and then successive screening was conducted according to the pre-set inclusion and exclusion criteria: first retaining English publications (n = 2846), then excluding non-original articles and non-reviews (retaining n = 2766), and finally conducting manual screening of research content relevance to exclude irrelevant documents, with 2633 valid publications retained for subsequent bibliometric and visual analysis.

The process of literature retrieval and screening was carried out independently by two authors. Any discrepancies were resolved through consultation with the third author. The literature search process for this study is illustrated in Figure 1.

### 2.2 Data analysis and visualization

CiteSpace and VOSviewer are widely utilized software tools for bibliometric and visual analysis (Miao B. et al., 2024; Tao et al., 2024). CiteSpace excels in extracting latent information from literature, enabling precise analysis of research hotspots and trends. Conversely, VOSviewer is renowned for its robust visualization capabilities, effectively presenting document knowledge maps and offering an intuitive overview of the entire research domain (Chen, 2004; van Eck and Waltman, 2010). The integration of these two tools offers researchers a comprehensive and efficient analytical approach, facilitating a deeper understanding of the current research landscape and future directions in the field of sepsis-related immunosuppression. The key parameter settings of CiteSpace were as follows: (Singer et al., 2026): Timespan: 2005–2025 (Slice Length = 1); (Rudd et al., 2020); Selection Criteria: g-index (k = 25), LRF = 2.5, L/N = 10, LBY = 5, e = 1.0; (Morrissey et al., 2025); Nodes Labeled: 1.0%; (Gao X. et al., 2025); Pruning: Pathfinder. The key parameter settings of VOSviewe were as follows: (Singer et al., 2026): Weights: Occurrences; (Rudd et al., 2020); Labels: Circles; (Morrissey et al., 2025); Font: Open Sans; (Gao X. et al., 2025); Max. length: 30.

## 3 Results

### 3.1 The publication trend

**FIGURE 2:**
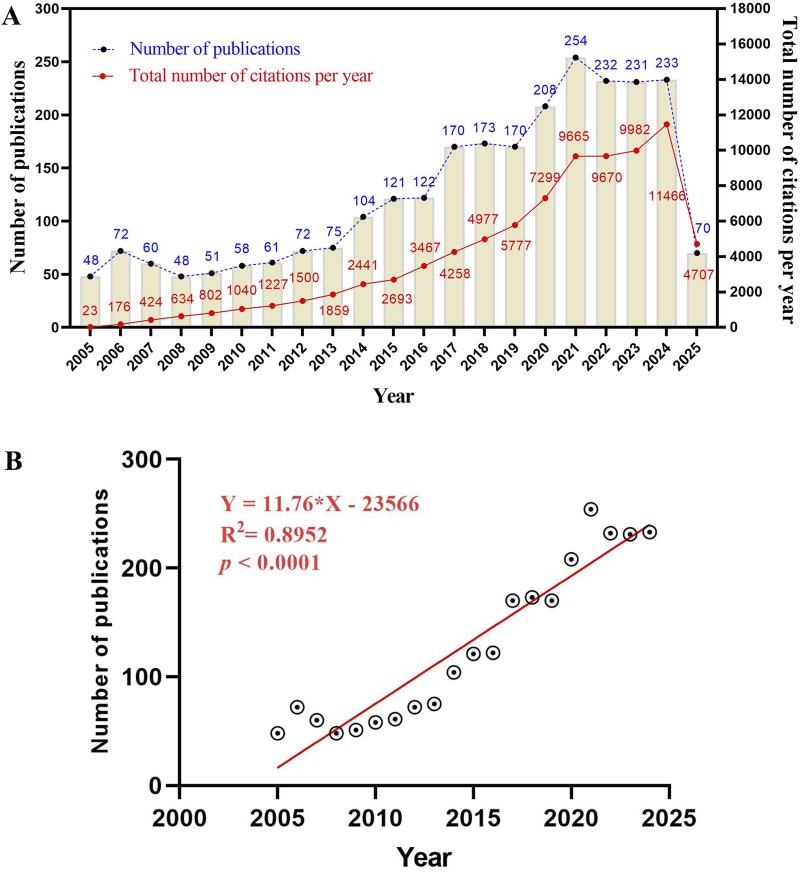
Annual publication trends in the field of sepsis-related immunosuppression. **(A)** Annual publication volume (blue line) and total annual citations (red line) of sepsis-related immunosuppression research from 2005 to 2024. The publication volume reflects the research output scale and development speed of the field in different periods, and the total citations represent the academic influence and attention degree of the field’s research results over time. **(B)** Linear fitting curve of the annual publication volume in this field, with the fitting equation Y = 11.76 *X – 23,566, the correlation coefficient *R* ^2^ = 0.8952, and *p* ≤ 0.0001, indicating a significant positive linear correlation between publication volume and research year.

A total of 2,633 articles on sepsis-related immunosuppression were included in this study. As illustrated in Figure 2A, the trajectory of publications in this domain exhibited distinct phases from 2005 to 2024. The period from 2005 to 2012 could be characterized as an initial growth phase, during which the number of publications gradually increased from 48 to 72, indicative of preliminary exploration in the field. Subsequently, from 2013 to 2021, the field experienced a phase of rapid expansion, with publication numbers rising from 75 to 254, reflecting heightened interest and engagement. The years 2022–2024 represented a plateau phase, with publication numbers stabilizing between 231 and 233, suggesting a potential bottleneck in research advancement. The publication trend is further elucidated by the fitting curve (y = 11.76 * x–23566, *R* ^2^ = 0.8952, *p* ≤ 0.0001) presented in Figure 2B, which demonstrates a significant positive correlation between the overall volume of publications and the year, thereby indicating a substantial temporal increase in publication output. The total number of citations has risen significantly from 23 in 2005 to 11,466 in 2024, indicating a continued growth in the influence of this research field. Despite a plateau in publication volume from 2022 to 2024, the overall long-term linear growth trend suggests that publication volume is likely to increase in the future once the current bottleneck is overcome.

### 3.2 Countries and institutions

**FIGURE 3:**
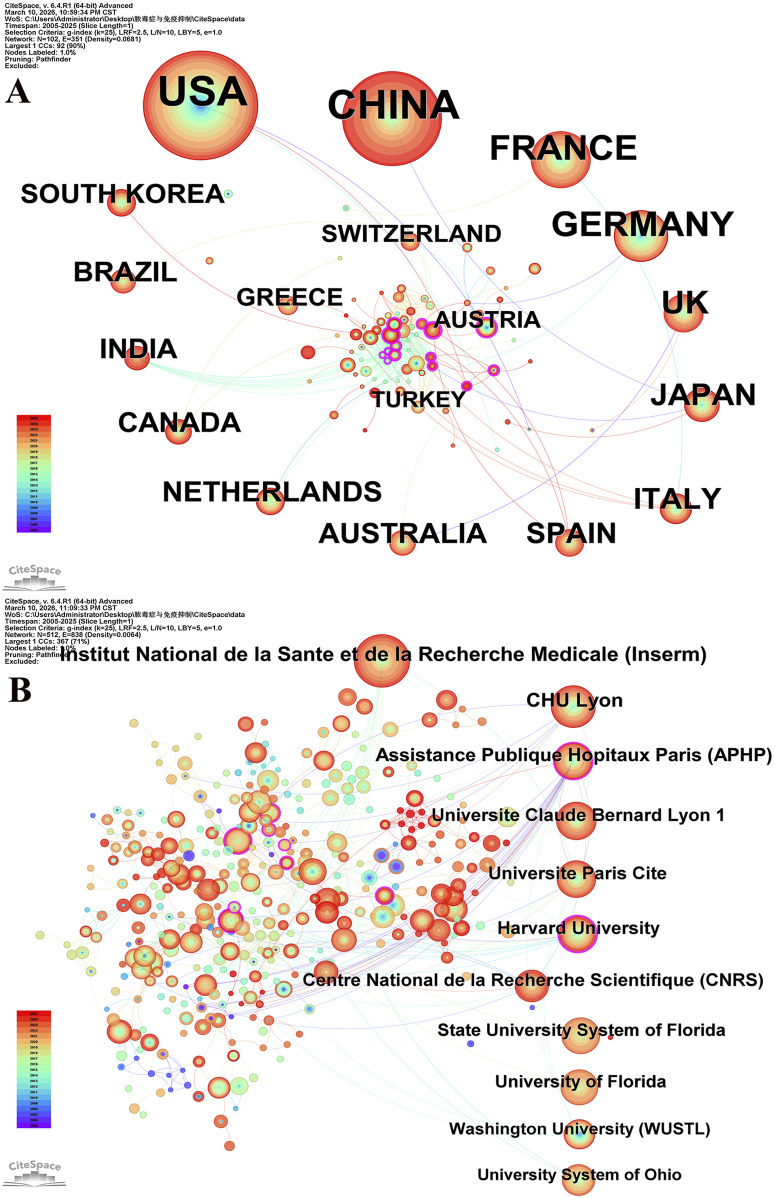
The co-occurrence map of countries **(A)** and institutions **(B)** in the field. Node size is positively correlated with the number of publications of countries/institutions (the larger the node, the higher the publication output); the presence of connecting lines between nodes indicates the existence of collaborative research relationships between the two parties; the clockwise arrangement of nodes in Panel A is based on the descending order of national publication volume. Panel A includes 102 countries, with the USA, China, and France as core contributors. Panel B focuses on institutions, highlighting major contributors and their collaborative networks. The map intuitively reflects the distribution of core research forces and the characteristics of international/inter-institutional collaboration in this field.

Numerous countries and scientific research institutions have made substantial contributions to this field of research. The United States was at the forefront with 776 publications, followed by China with 638 publications. France (n = 243), Germany (n = 229), and the United Kingdom (n = 150) were also prominent contributors to the publication landscape (refer to Table 1). In terms of centrality, Bahrain (0.30), South Africa (0.29), and Belarus (0.28) ranked highly, indicating their pivotal role as intermediaries in this domain. At the institutional level, the Institut National de la Santé et de la Recherche Médicale (INSERM) leads with 134 published articles, followed by CHU Lyon (n = 101) and Assistance Publique–Hôpitaux de Paris (APHP) (n = 84) (see Table 1). The substantial volume of publications from these countries and institutions underscores their significant investment of research resources and high level of research activity in this field. Figure 3 illustrates the increasing frequency of collaborations between countries and institutions in recent years. These cooperations have played an important role in promoting the development and progress in the field.

**TABLE 1:** Top 10 contributing countries, institutions, journals, and cited journals in sepsis-related immunosuppression research.

Category	Rank	Entity	Metric	Count	Centrality
Country	1	USA	Publications	776	0.07
	2	CHINA	Publications	638	0
	3	FRANCE	Publications	243	0.04
	4	GERMANY	Publications	229	0
	5	UK	Publications	150	0.04
	6	JAPAN	Publications	114	0.07
	7	ITALY	Publications	95	0.07
	8	SPAIN	Publications	85	0
	9	AUSTRALIA	Publications	78	0.07
	10	NETHERLANDS	Publications	78	0.04
Institution	1	Institut National de la Sante et de la Recherche Medicale (Inserm)	Publications	134	0.07
	2	CHU Lyon	Publications	101	0.09
	3	Assistance Publique Hopitaux Paris (APHP)	Publications	84	0.15
	4	Universite Claude Bernard Lyon 1	Publications	72	0
	5	Universite Paris Cite	Publications	72	0.04
	6	Harvard University	Publications	68	0.21
	7	Centre National de la Recherche Scientifique (CNRS)	Publications	66	0.07
	8	State University System of Florida	Publications	62	0
	9	University of Florida	Publications	59	0
	10	Washington University (WUSTL)	Publications	49	0.01
Journal (by output)	1	Frontiers in Immunology	Publications	139	—
	2	Shock	Publications	109	—
	3	PLoS One	Publications	68	—
	4	Critical Care	Publications	64	—
	5	Transplantation Proceedings	Publications	60	—
	6	Critical Care Medicine	Publications	58	—
	7	Journal of Immunology	Publications	40	—
	8	International Immunopharmacology	Publications	38	—
	9	Journal of Leukocyte Biology	Publications	31	—
	10	Scientific Reports	Publications	31	—
Journal (by citation)	1	Critical Care	Citations	4140	—
	2	Frontiers in Immunology	Citations	3,880	—
	3	Critical Care Medicine	Citations	3,508	—
	4	Shock	Citations	3,229	—
	5	Journal of Immunology	Citations	2239	—
	6	Intensive Care Medicine	Citations	1998	—
​	7	PLoS One	Citations	1903	—
	8	Journal of Clinical Investigation	Citations	1452	—
	9	Journal of Leukocyte Biology	Citations	1419	—
	10	American Journal of Respiratory and Critical Care Medicine	Citations	1411	—

This table systematically summarizes the top 10 contributors in four core dimensions of sepsis-related immunosuppression research from 2005 to 2025, including publication output of countries and institutions, and publication volume/citation frequency of journals. The “Centrality” index reflects the intermediary role of countries/institutions in the global research collaboration network (higher values indicate stronger bridging and collaborative radiation capacity); for journal metrics, publication volume represents the research output capacity, while citation frequency reflects the academic influence and recognition of journals in this field.

### 3.3 Journals

**FIGURE 4:**
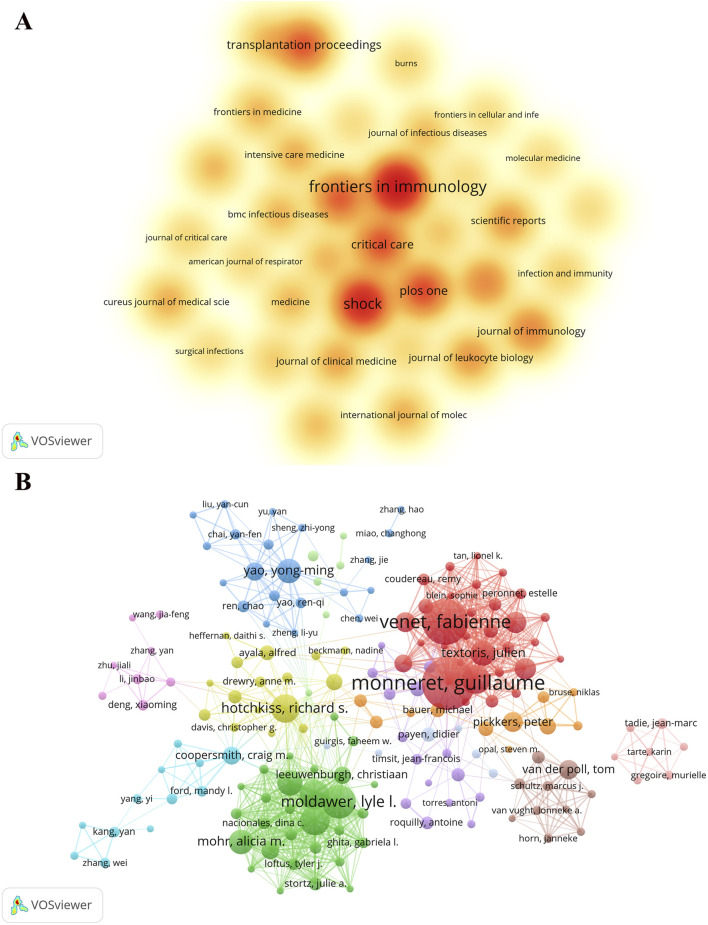
The density map of journals **(A)** and the collaboration network map of authors **(B)** in the field. **(A)** The intensity of color is positively correlated with the frequency of journal publications in this domain, indicating that darker colors signify a higher volume of research articles on sepsis-related immunosuppression published within these journals. This serves to highlight the principal academic journals and primary channels for research dissemination in this area. **(B)** The size of the nodes denotes the number of publications attributed to individual authors, with larger nodes indicating a higher publication output. The lines connecting the nodes illustrate co-authorship relationships, while the distinct color clusters of nodes represent independent core research teams within this field. The map clearly shows the prolific journals, researchers, and core research teams.

The documents on sepsis-related immunosuppression included in this study were published in 870 academic journals, indicating extensive attention to this research field. Regarding the number of published documents, *Frontiers in Immunology* ranked first with 139 papers, followed by Shock (n = 109), *PLoS One* (n = 68), and *Critical Care* (n = 64) (Table 1). Figure 4A intuitively represents these high-output journals. These publications are not only pivotal in the domains of sepsis and immunology but also underscore the interdisciplinary nature of this research field. Further analysis of citation frequency reveals that *Critical Care* ranks first with 4,140 cited times, followed by *Frontier in immunology* (n = 3,880) and *Critical Care Medicine* (n = 3,508) (Table 1). These highly cited journals are not only well-regarded in academic circles but also serve as valuable resources for advancing the study of sepsis-related immunosuppression.

### 3.4 authors

Statistics show that 14,897 authors have co-authored these publications. In terms of the volume of papers published, Guillaume Monneret ranked first with 84 papers, followed by Fabienne Venet (n = 69) and Lyle L. Molddawer (n = 44) (Table 2). Furthermore, the author collaboration network comprises approximately 10 major research groups (Figure 4B). The cited author’s analysis further reveals the influential scholars in the field. According to the data, Richard S. Hotchkiss ranked first with 7,584 citations, followed by Guillaume Monet (n = 6,797) and Fabienne Venet (n = 3,492) (Table 2). Future research can focus on the latest achievements of these scholars to track the Frontier directions in this research field.

**TABLE 2:** The top 10 authors in this research field.

Rank	Author	Documents	Cited author	Citations
1	Guillaume Monneret	84	Richard S. Hotchkiss	7584
2	Fabienne Venet	69	Guillaume Monneret	6797
3	Lyle L. Moldawer	44	Fabienne Venet	3,492
4	Philip A. Efron	37	Didier Payen	3,337
5	Frederick A. Moore	37	Lyle L. Moldawer	3,140
6	Richard S. Hotchkiss	35	Frederick A. Moore	2645
7	Thomas Rimmele	35	Philip A. Efron	2506
8	Scott C. Brakenridge	30	Scott C. Brakenridge	2074
9	Julien Textoris	29	Andrew H. Walton	2015
10	Alicia M. Mohr	28	Jonathan M. Green	1952

This table systematically summarizes the top 10 contributors in four core dimensions of sepsis-related immunosuppression research from 2005 to 2025, including publication output of countries and institutions, and publication volume/citation frequency of journals. The “Centrality” index reflects the intermediary role of countries/institutions in the global research collaboration network (higher values indicate stronger bridging and collaborative radiation capacity); for journal metrics, publication volume represents the research output carrying capacity, while citation frequency reflects the academic influence and recognition of journals in this field.

### 3.5 The keyword analysis

**FIGURE 5:**
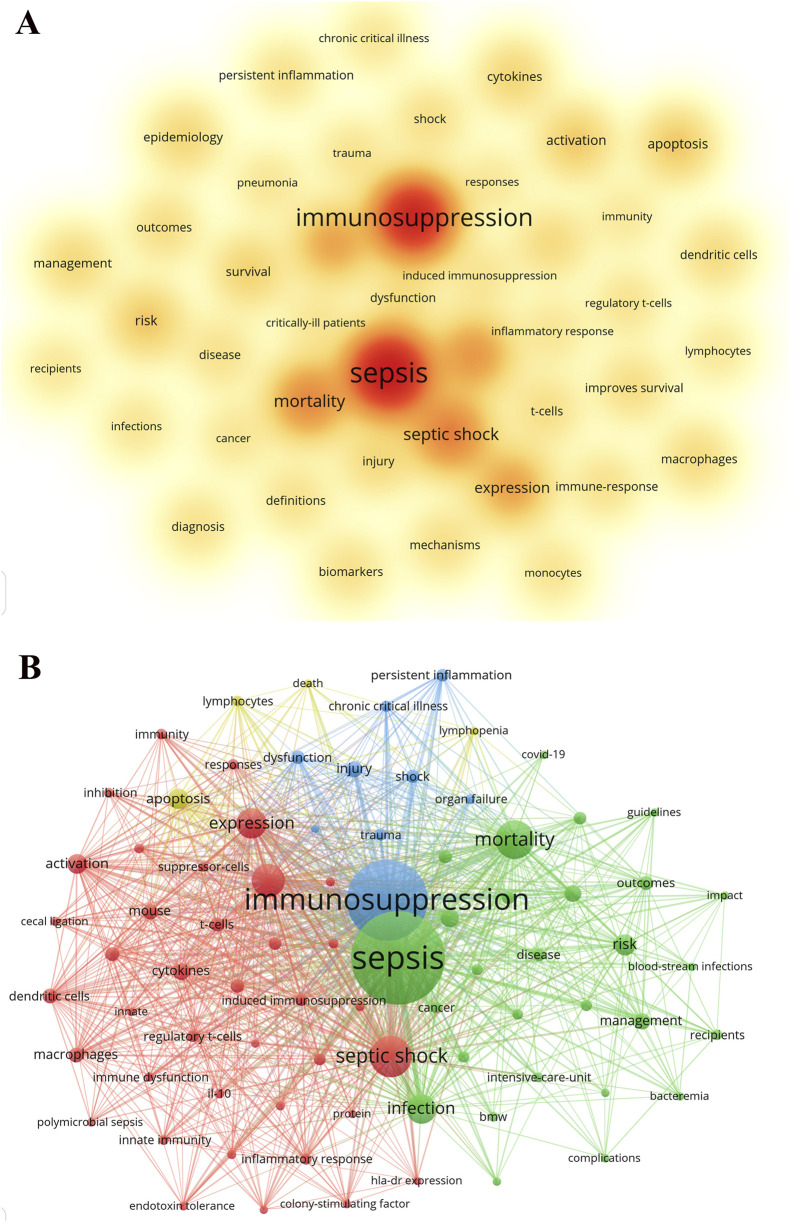
The co-occurrence density map **(A)** and network **(B)** of keywords about the field. **(A)** Keyword co-occurrence density map, where color intensity is positively correlated with the co-occurrence frequency of keywords, intuitively showing the core research terms and their concentration in this field (e.g., sepsis, immunosuppression, septic shock). **(B)** Keyword co-occurrence network map, where node size represents the occurrence frequency of keywords; connecting lines indicate co-occurrence relationships between keywords; different color clusters represent different research directions and sub-fields divided by cluster analysis. Four main research clusters were identified in the map, with representative keywords of septic shock, sepsis, immunosuppression, and apoptosis, respectively, reflecting the main research branches and the internal connection between different research themes in this field. **(A)** Minimum number of occurrences of keywords ≥70; **(B)** Minimum number of occurrences of keywords ≥50. Keyword clusters with different colors represent different research directions.

Following the cleaning of the original dataset and the consolidation of synonyms, a total of 8,265 keywords were identified in this study. As illustrated in Table 3, the keyword with the highest frequency is “sepsis” (n = 1,549), followed by “immunosuppression” (n = 1,262) and “septic shock” (n = 486). Figure 5A provides a more intuitive representation of these high-frequency keywords. These keywords encompass a broad spectrum of research interests within the domain of sepsis-related immunosuppression, ranging from fundamental studies on immune mechanisms to clinical management and treatment strategies, as well as epidemiology and prognostic assessments. The visual keyword co-occurrence network depicted in Figure 5B allows for a clear observation of the interrelationships among keywords. Through cluster analysis, four primary clusters were identified. The representative keyword for Cluster 1 (red) is “septic shock”, while Cluster 2 (green) is characterized by “sepsis”. Cluster 3 (blue) is represented by “immunosuppression”, and Cluster 4 (yellow) is denoted by “apoptosis.”

**TABLE 3:** Top 20 keywords related to this research field.

Rank	Keyword	Counts	Rank	Keyword	Count
1	sepsis	1549	11	survival	152
2	immunosuppression	1262	12	cytokines	140
3	septic shock	486	13	epidemiology	135
4	mortality	441	14	management	128
5	inflammation	355	15	injury	126
6	expression	309	16	mouse	119
7	infection	299	17	macrophages	117
8	risk	183	18	dendritic cells	116
9	apoptosis	180	19	improves survival	109
10	activation	174	20	outcomes	109

**FIGURE 6:**
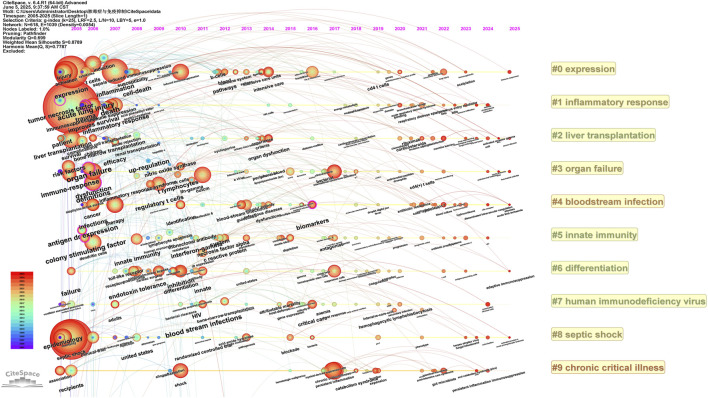
The timeline viewer of keywords about the field. It covers the time range from 2005 to 2024, showing the distribution of keywords in the field of sepsis-related immunosuppression with time. Each node represents a keyword, with node size positively correlated with the occurrence frequency of the keyword; connecting lines between nodes indicate the co-occurrence relationship between keywords; different colors of nodes correspond to the publication year of the documents where the keywords first appeared in large quantities; the 10 clusters marked with #0 to #9 represent the main research sub-fields formed in the development of the field. The map dynamically shows the evolution of research hotspots, the emergence of new research keywords, and the transformation of research focus in sepsis-related immunosuppression research over time, and can intuitively reflect the stage characteristics of the field’s research development (e.g., the initial focus on basic correlation research, the intermediate focus on mechanism exploration, and the recent dispersed research directions).

The timeline viewer of keywords (Figure 6) effectively illustrates the dynamic evolution of the research field by depicting the distribution, frequency, and co-occurrence relationships of keywords across different years. For instance, years characterized by a dense distribution of keywords often align with periods of rapid advancement in the field, potentially marked by significant theoretical breakthroughs or technological innovations. Conversely, periods of sparse keyword distribution may indicate that the research field has entered a plateau or is undergoing a phase of directional adjustment.

### 3.6 Co-cited articles and reference burst

In bibliometrics, co-citation literature analysis is a method used to reveal the correlation between documents by investigating situations in which multiple documents are cited by other documents at the same time. When many documents are cited by other documents at the same time, it shows that these documents are similar or related. These core documents are usually classic studies or important achievements in this field, which can help researchers quickly understand the research foundation and important achievements in this field. Through the co-citation analysis of the literature, we screened out several highly co-cited articles (Table 4). Among them, the study “Immunosuppression in patients who die of separation and multiple organ failure” published by Boomer et al. (2011) in 2011 was co-cited the most, with a total of 125 times. Boomer et al. (2011) demonstrated that patients with sepsis exhibited pronounced immunosuppression at the time of death by comparing the immune status of patients with severe sepsis to those who succumbed to non-septic conditions.

**TABLE 4:** The top 10 co-cited articles related to the research field.

Rank	Author	Title	Journal	Year	Co-citations	Centrality
1	Boomer et al. (2011)	Immunosuppression in patients who die of sepsis and multiple organ failure	Journal of the American Medical Association	2011	125	0.01
2	Francois et al. (2018)	Interleukin-7 restores lymphocytes in septic shock: the IRIS-7 randomized clinical trial	JCI Insight	2018	74	0.06
3	Mathias et al. (2017)	Human Myeloid-derived Suppressor Cells are Associated with Chronic Immune Suppression After Severe Sepsis/Septic Shock	Annals of Surgery	2017	57	0.06
4	Hotchkiss et al. (2019a)	Immune Checkpoint Inhibition in Sepsis: A Phase 1b Randomized, Placebo-Controlled, Single Ascending Dose Study of Antiprogrammed Cell Death-Ligand 1 Antibody (BMS-936559)	Critical Care Medicine	2019	56	0.02
5	Hotchkiss et al. (2019b)	Immune checkpoint inhibition in sepsis: a Phase 1b randomized study to evaluate the safety, tolerability, pharmacokinetics, and pharmacodynamics of nivolumab	Intensive Care Medicine	2019	50	0.01
6	Stortz et al. (2018)	Evidence for Persistent Immune Suppression in Patients Who Develop Chronic Critical Illness After Sepsis	Shock	2018	45	0.02
7	Drewry et al. (2014)	Persistent lymphopenia after diagnosis of sepsis predicts mortality	Shock	2014	44	0.01
8	Uhel et al. (2017)	Early Expansion of Circulating Granulocytic Myeloid-derived Suppressor Cells Predicts Development of Nosocomial Infections in Patients with Sepsis	American Journal of Respiratory and Critical Care Medicine	2017	44	0.01
9	Chang et al. (2014)	Targeting the programmed cell death 1: programmed cell death ligand 1 pathway reverses T cell exhaustion in patients with sepsis	Critical Care	2014	43	0.06
10	Patera et al. (2016)	Frontline Science: Defects in immune function in patients with sepsis are associated with PD-1 or PD-L1 expression and can be restored by antibodies targeting PD-1 or PD-L1	Journal of Leukocyte Biology	2016	40	0.02

Co-citation frequency refers to the number of times two documents are cited together by other documents, and highly co-cited articles are the classic foundational studies and key breakthrough achievements in this field, which have laid an important foundation for the development of subsequent research.

**FIGURE 7:**
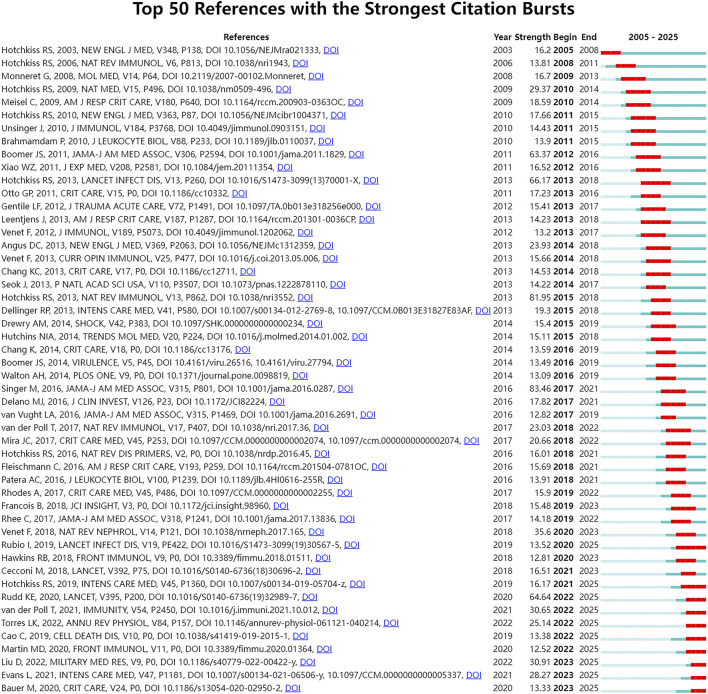
Top 50 references with the strongest citation bursts. Citation burst refers to the sudden significant increase in the citation frequency of a document over a specific period, which is an important indicator for identifying emerging research topics and influential breakthrough studies in the field. The chart lists the core information of each burst reference, including author, publication year, journal, DOI, burst strength (the higher the value, the more significant the sudden increase in citations), burst start and end time; the blue bar represents the publication period of the reference, and the red bar represents the citation burst period of the reference. The map can clearly identify the classic documents that have had a key impact on the development of the field in different periods and the emerging research literature that have attracted extensive attention in recent years, and reveal the evolution of the core research foundation and frontier hotspots of the field.

Sudden citation analysis can quickly identify frequently cited documents in a certain period, which usually represent a new research topic in this field. In this study, we employed the sudden citation analysis method to detect emergent literature (Figure 7) by examining temporal fluctuations in citation frequency. Our analysis identified 10 articles that have attracted substantial attention, accounting for 20% of the total publications analyzed. These documents have garnered significant attention in a brief period, potentially signifying a novel research focus in the area of sepsis-related immunosuppression.

### 3.7 The verification based on the PubMed database

To further validate the stability of this study’s findings, we conducted an independent analysis using multiple databases. This approach aimed to demonstrate that the research conclusions are not reliant on a single data source but are instead universally applicable. The PubMed database, which predominantly comprises documents from the biomedical field and sources data from biomedical journals worldwide, is recognized as one of the most authoritative literature databases in this discipline. Given that the subject of this study falls within the medical field, we supplemented our research with relevant literature from the PubMed database for bibliometric analysis. A total of 550 reviews and articles were included in the analysis.

**FIGURE 8:**
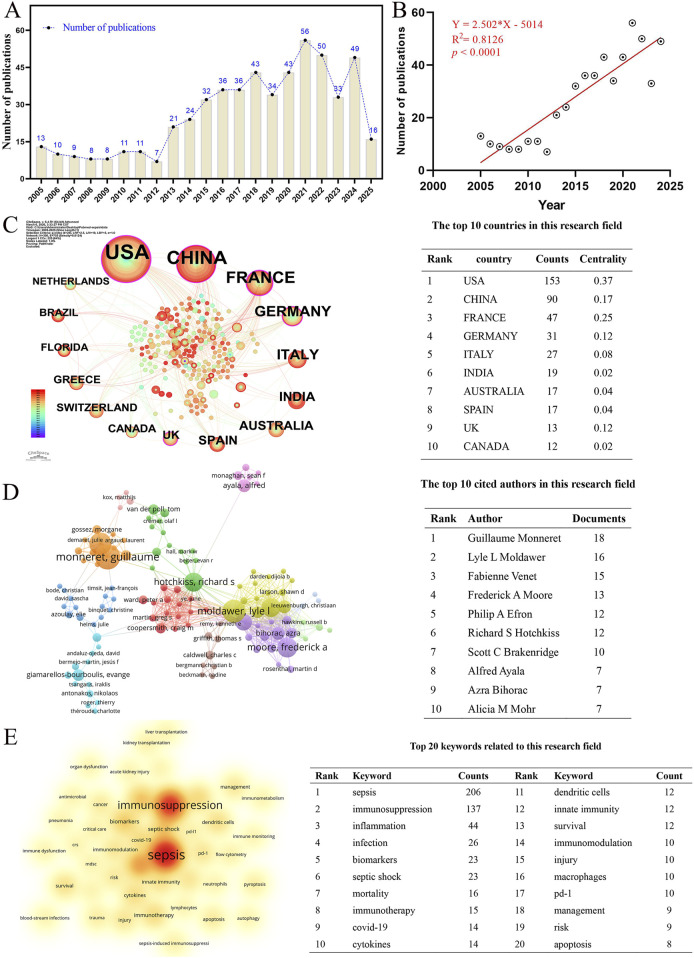
Based on the verification results from the PubMed database Comprehensive verification results of sepsis-related immunosuppression research based on the PubMed database (a total of 550 valid English original articles and reviews were included after screening), including **(A)** annual publication trend of the field in the PubMed database, **(B)** linear fitting curve of publication volume (fitting equation Y = 2.502 *X – 5014, *R* ^2^ = 0.8126, *p* ≤ 0.0001), **(C)** top 10 contributing countries by publication volume, **(D)** top 10 contributing authors by publication volume, and **(E)** top 10 high-frequency keywords.

The findings indicate that, with respect to publishing trends, the number of publications in this field exhibits phases of stability, rapid expansion, and a recent plateau period (Figure 8A). The publication trend is further elucidated by the fitting curve (Y = 2.502 * X–5014, *R* ^2^ = 0.8126, *p* ≤ 0.0001) (Figure 8B), which demonstrates a significant positive correlation between the total volume of publications and the year, suggesting an increase in publication volume over time. On a national scale, the United States emerges as the most prolific contributor, with 153 publications, followed by China (n = 90) and France (n = 47) (Figure 8C). At the author level, Guillaume Monneret is the leading contributor with 18 publications, followed by Lyle L. Moldawer (n = 16) and Fabienne Venet (n = 15) (Figure 8D). Regarding keyword frequency, “sepsis” (n = 206) is the most prevalent, followed by “immunosuppression” (n = 137) and “information” (n = 44) (Figure 8E).

The bibliometric analysis results from the PubMed database are very similar or overlap significantly with those from the WoSCC database. For instance, when examining the annual publication trends, the trends of the two were remarkably similar (Figures 1A, 8A). The correlation coefficients *R* ^2^ for their publication fitting curves were also notably close, being 0.8952 and 0.8126, respectively (Figures 1B, 8B). The overlap rate among the top 10 countries was 80%, as indicated in Table 1 and Figure 8C. Similarly, the overlap rate among the top 10 researchers was also 80%, as shown in Table 3 and Figure 8D. Furthermore, the overlap rate for the top 20 keywords by frequency was 70%. In summary, the bibliometric analysis conducted using the PubMed database corroborates the stability and reliability of the results of this study.

## 4 Discussion

Sepsis is characterized as a syndrome of multiple organ dysfunction resulting from infection, and its pathophysiological mechanisms are intricate, involving both the hyperactivation of the immune system and subsequent immunosuppression (Gao X. et al., 2025). Patients experiencing sepsis accompanied by immunosuppression face a markedly elevated risk of secondary infections and mortality (Gao X. et al., 2025). Investigating sepsis-related immunosuppression is not only instrumental in elucidating the pathogenesis of sepsis but also holds significant implications for guiding clinical interventions and enhancing patient prognosis. Utilizing bibliometric and visualization methodologies, this study systematically examines global research trends and focal areas concerning sepsis-related immunosuppression from 2005 to 2025, thereby offering novel insights and perspectives for future research in this domain.

Recently, Yin et al. (2025) conducted a bibliometric analysis that offered a comprehensive overview of research on sepsis-related immunosuppression spanning from 2004 to 2024. In contrast, our study investigates this domain from alternative perspectives, encompassing the period from 2005 to 2025. We employ a more targeted search strategy, incorporating independent verification through the PubMed database, and integrate keyword analysis with citation burst detection to identify emerging topics. These methodological improvements, combined with the updated data, have provided a unique interpretation for this research field, enabling scholars to grasp this evolving field from multiple perspectives.

Furthermore, numerous meta-analyses have been conducted to investigate specific aspects of sepsis-related immunosuppression, yielding valuable insights. For instance, Schaack et al. (2018) performed a comprehensive microarray meta-analysis to examine the heterogeneity of immune responses in early sepsis and to identify gene expression characteristics associated with immunosuppression. Similarly, a recent meta-analysis by García-Aguilera et al. (2025) focused on the mortality rates of cancer patients experiencing septic shock in intensive care units, providing crucial data for the prognosis of high-risk subgroups. This research underscores the clinical significance of sepsis-related immunosuppression within a specific population. These two meta-analyses delve into the mechanistic and clinical intricacies, whereas the present study offers a holistic overview of the field. We posit that these two approaches are complementary and contribute to shaping future research directions and fostering interdisciplinary integration.

### 4.1 General information

The trajectory of publications in this field exhibits a distinct three-phase pattern: an initial phase from 2005 to 2012, a period of rapid growth from 2013 to 2021, and a plateau phase from 2022 to 2024. During the initial phase (2005–2012), the publication rate was relatively slow, reflecting the nascent acknowledgment within academic circles of sepsis-induced immunosuppression as an independent pathogenic factor. Before this period, research on sepsis predominantly concentrated on the “cytokine storm” associated with inflammation, with immunosuppression being considered a secondary, late-stage complication rather than a primary driver of mortality (Boomer et al., 2011). The limited number of publications at this stage can primarily be attributed to two key factors. First, the absence of standardized diagnostic criteria for sepsis-induced immunosuppression impedes the reproducibility of studies and the comparison of cohorts. Second, the scarcity of targeted experimental models for investigating immune cell dysfunction in sepsis constrains the exploration of underlying mechanisms. Notably, the landmark study by Boomer et al. (2011), which demonstrated significant immunosuppression in patients with severe sepsis, marked a pivotal turning point. This study laid the groundwork for the subsequent rapid growth in this research area and confirmed the clinical relevance of this line of inquiry.

During the period of rapid growth from 2013 to 2021, the number of published papers increased significantly, rising from 75 to 254. This surge may be attributed to the convergence of foundational immunology, clinical sepsis research, and global investment in scientific research. Several factors contribute to this phenomenon: First, the release of the Sepsis-3 definition in 2016 standardized the diagnostic criteria for septicemia, thereby enhancing patient stratification accuracy and reducing heterogeneity in clinical research related to immunosuppression (Singer et al., 2016). Second, significant breakthroughs in immunology, including the identification of the PD-1/PD-L1 axis and MDSCs as critical regulatory factors in sepsis-induced immunosuppression, have paved the way for new mechanisms and therapeutic research avenues (Zhang T. et al., 2023; Xiang et al., 2025).

During the platform period of 2022–2024, the annual number of publications remained stable, ranging from 231 to 233 articles, a trend that warrants critical examination. Several factors may contribute to this phenomenon. First, the research in this domain predominantly concentrates on established pathways and immune cell subgroups, with limited breakthroughs in identifying novel regulatory networks, thereby decelerating overall research progress. Second, the clinical translation of research findings in this field poses significant challenges, impeding its development. The immune response in sepsis patients is temporally dynamic, encompassing multiple pro-inflammatory and anti-inflammatory mechanisms (Venet and Monneret, 2018). This complexity complicates clinical trial design, as the variability in patients’ immune status and biomarker heterogeneity hinders the development of personalized treatments (Rao et al., 2025). Moreover, the immune status of sepsis patients not only varies between individuals but also fluctuates over the course of the disease in the same patient, further exacerbating the challenges of clinical translation (Rao et al., 2025). Third, the COVID-19 pandemic reallocated substantial research funding and personnel from septicemia to the emergency response for infectious diseases, likely postponing the initiation of new projects and the publication of ongoing research (Raynaud et al., 2021).

The United States has emerged as the leading contributor in terms of publication output, followed by China and France. This trend underscores the United States’ prominent role in both research input and output within this field, likely facilitated by its robust research infrastructure, ample funding, and abundant resources. China’s growing focus and investment in this area have also led to a notable increase in its publication output. Among research institutions, the Institut National de la Santé et de la Recherche Médicale (INSERM) in France has produced the highest number of publications, underscoring its significant expertise in this field. Furthermore, 60% of the top ten research institutions are based in France, with the remaining 40% located in the United States, further affirming the dominant positions of these two countries in sepsis-related immunosuppression research. This concentration of leading institutions may reflect their advantages in scientific collaboration, resource integration, and research innovation, enabling them to maintain a leading position and consistently produce high-quality research outcomes. It is worth noting that French institutions have shown great research strength in this field, which may be related to France’s traditional advantages in immunology and critical care medicine. Richard S. Hotchkiss and Guillaume Monneret are the most cited researchers. *Critical Care* and *Frontiers in Immunology* are journals that are frequently cited. This may mean that their research has an important influence and recognition in this research field, and their work may represent the research Frontier and hotspot in this field.

### 4.2 The research direction and research hotspot analysis

Table 3 delineates the principal research themes and their progression within the domain of sepsis-related immunosuppression. The frequent occurrence of the keywords “sepsis” and “immunosuppression” underscores the foundational focus of research in this area, while the prominence of “septic shock” and “mortality” highlights the significant concern for clinical severity and patient prognosis. Notably, the recurrent mention of “apoptosis,” “dendritic cells,” and “macrophages” suggests that the molecular mechanisms underlying immune cell death and dysfunction have emerged as a critical area of investigation. Concurrently, the prevalence of “cytokines” and “activation” underscores the pivotal role of inflammatory mediator regulation and the assessment of immune activation. Furthermore, the introduction of terms such as “management” and “improvements in survival” signifies a shift from fundamental mechanistic research towards the development of clinical treatment strategies. Collectively, the distribution of these keywords not only reflects the current comprehensive exploration of the pathological mechanisms underlying immunosuppression but also indicates a future trajectory towards precision immunotherapy guided by biomarkers.

Cluster analysis can classify similar or related keywords into one category, thus identifying the core topics and hot issues in the research field. Utilizing a clustering analysis algorithm, the keywords relevant to this field have been organized into four distinct clusters (Figure 5B), each potentially representing a principal research trajectory within the domain. These four research trajectories encompass: 1) the mechanisms underlying sepsis-related immunosuppression, including the dysfunction of immune cells such as dendritic cells, macrophages, and T cells, as well as the regulation of cytokines including IL-10, IFN-γ, and TNF; 2) the epidemiological characteristics, diagnostic criteria, clinical management, and prognostic evaluation of sepsis, along with its manifestations and impacts in specific populations, including pediatric patients, critically ill individuals, and those co-infected with COVID-19; 3) the relationship between organ damage and dysfunction mediated by sepsis-induced immunosuppression; and 4) a cluster characterized by a limited number of keywords, potentially associated with lymphocyte apoptosis and reduction in sepsis-related immunosuppression, which may elucidate the role of immune cell death mechanisms in immunosuppression.

To further investigate the research focal points within the domain of “sepsis-related immunosuppression,” this study performed a co-citation analysis of the literature about this field, identifying the top 10 articles based on co-citation frequency (Boomer et al., 2011; Francois et al., 2018; Mathias et al., 2017; Hotchkiss et al., 2019a; Hotchkiss et al., 2019b; Stortz et al., 2018; Drewry et al., 2014; Uhel et al., 2017; Chang et al., 2014; Patera et al., 2016). A comprehensive examination of these articles revealed the following key research areas in sepsis-related immunosuppression.

Investigation into the mechanisms underlying immune cell dysfunction, specifically involving T cells and monocytes, as well as the reduction in cytokine secretion following sepsis (Boomer et al., 2011).

The impairment of immune cell function and the reduction in cytokine secretion following sepsis represent a complex process, characterized by the interplay of numerous cellular and molecular mechanisms. The post-sepsis alteration in immune cell function is attributed to various factors, including cellular metabolic reprogramming, increased apoptosis, and the modulation of immune checkpoint molecules. Firstly, the functional impairment of CD4^+^ T cells post-sepsis is associated with metabolic reprogramming (Assis et al., 2024). Research indicates that sepsis can induce mitochondrial dysfunction and glycolysis in CD4^+^ T cells, with these metabolic alterations initiating during acute sepsis and persisting thereafter, consequently impacting the immune response of T cells to subsequent infections (Assis et al., 2024). Secondly, monocytes exhibit prolonged dysfunction following sepsis, which is associated with the persistent activation of macrophage colony-stimulating factor (M-CSF). Research indicates that post-sepsis, monocytes lose their adaptive capacity, and their ability to differentiate into dendritic cells diminishes, a phenomenon linked to the sustained secretion of M-CSF and the demethylation of PU.1 (Lapko et al., 2017). Furthermore, the upregulation of immune checkpoint molecules, such as TIGIT and PD-1, subsequent to sepsis, can also result in T cell dysfunction. Evidence suggests that the expression of TIGIT correlates with the exacerbation of inflammatory responses and organ damage in sepsis patients, and its expression on CD8^+^ T cells can predict the mortality of intensive care unit (ICU) patients (Sun Y. et al., 2021). Additionally, sepsis can lead to increased apoptosis of immune cells, particularly T cells. Studies have demonstrated that inhibiting autophagy in T cells accelerates their apoptosis, resulting in a reduction in T cell numbers and compromised function, thereby elevating sepsis-related mortality (Oami et al., 2017). Finally, the decrease of cytokine secretion after sepsis is also an important feature of immunosuppression. Research has demonstrated a marked decrease in the production of cytokines, including TNF-α and IL-6, by peripheral blood mononuclear cells in patients with sepsis. This immunosuppressive state can persist for several days and is associated with adverse prognostic outcomes (Antonakos et al., 2017). Thorough exploration of the mechanisms driving post-sepsis immunosuppression is crucial for providing a theoretical basis for the development of innovative therapeutic approaches.

Investigation into the mechanisms by which the PD-1/PD-L1 pathway facilitates immunosuppression associated with sepsis (Chang et al., 2014; Patera et al., 2016).

The PD-1/PD-L1 pathway is integral to the immunosuppressive mechanisms associated with sepsis. PD-L1, a co-inhibitory molecule expressed on immune cells, has been increasingly recognized as a critical factor in the pathophysiology of sepsis (Sari and Ilyas, 2022). Research indicates a significant correlation between the expression of PD-1/PD-L1 and sepsis, with particular emphasis on the expression of PD-L1 on neutrophils (Chen et al., 2023). During the initial stages of sepsis, there is a marked upregulation of PD-L1 in MDSCs and their subsets, which subsequently suppresses T cell proliferation via the PD-L1/PD-1 axis, contributing to immunosuppression (Ruan et al., 2020). Furthermore, the study has demonstrated that activation of the PD-1/PD-L1 pathway results in increased T-cell apoptosis and immune dysfunction, ultimately culminating in sepsis-induced immunosuppression (Liu et al., 2024). In clinical practice, ICIs targeting the PD-1/PD-L1 pathway have demonstrated success in cancer immunotherapy, and their potential application in the treatment of sepsis is anticipated. Nonetheless, the complexity and heterogeneity inherent in sepsis present significant challenges for clinical trials. Identifying specific subsets of immune paralysis that are amenable to PD-1/PD-L1 pathway inhibition is crucial (Nakamori et al., 2020). The role of the PD-1/PD-L1 pathway in the immunosuppressive mechanisms of sepsis is intricate, and further investigation into this pathway may yield novel therapeutic approaches for sepsis management.

Investigation into the immunosuppressive mechanisms facilitated by myeloid-derived suppressor cells (MDSCs) and low-density neutrophils (LDNs) (Mathias et al., 2017; Uhel et al., 2017; Patera et al., 2016).

In recent years, the immunosuppressive mechanisms of MDSCs and LDNs in sepsis have garnered considerable academic interest. MDSCs represent a heterogeneous population of myeloid cells characterized by their potent immunosuppressive capabilities, which enable them to inhibit the functions of T cells and other immune cells through various mechanisms (Li et al., 2025). In individuals with sepsis, there is a significant increase in the number of MDSCs (Zhang W. et al., 2023). These cells play a crucial role in the immune response associated with sepsis by suppressing both innate and adaptive immune responses (Zhang W. et al., 2023). For instance, MDSCs can exert their immunosuppressive effects by secreting substantial amounts of immunosuppressive molecules, such as IL-10, thereby contributing to disease persistence (Yaseen et al., 2020). Moreover, during the early stages of sepsis, the activation of MDSCs may facilitate immunosuppression *via* the PD-L1/PD-1 axis (Ruan et al., 2020). The involvement of LDNs in sepsis has garnered significant scholarly interest. In patients with sepsis, LDNs exhibit elevated expression of PD-L1, which may suppress T cell proliferation *via* the PD-L1/PD-1 axis, thereby contributing to immunosuppression (Ruan et al., 2020). Furthermore, LDNs are implicated in secondary infections and T-cell inhibition, primarily through PD-L1 expression (Charoensappakit et al., 2025). The immunosuppressive effects of MDSCs and LDNs in sepsis are also thought to be intricately linked to their metabolic pathways. For instance, MDSCs demonstrate heightened glycolytic activity, which not only supplies energy but also generates metabolites that enhance their immunosuppressive functions (Goldmann and Medina, 2024). Additionally, fatty acid metabolic pathways, such as fatty acid oxidation (FAO), are considered crucial in modulating the inhibitory activity of MDSCs (Goldmann and Medina, 2024). In summary, the immunosuppressive mechanisms of MDSCs and LDNs in sepsis are complex and multifaceted, involving a range of cellular signaling and metabolic pathways. A comprehensive investigation of these mechanisms holds promise for identifying novel biomarkers and therapeutic targets in the clinical management of sepsis.

To investigate the link between sepsis-related immunosuppression biomarkers (persistent lymphopenia, mHLA-DR, and sPD-L1 levels) and immunosuppression status and prognosis (Stortz et al., 2018; Drewry et al., 2014; Chang et al., 2014).

The immunosuppression state of sepsis patients is closely related to many biomarkers, which can be used to evaluate the immune state and prognosis of these individuals. Firstly, persistent lymphopenia serves as a critical indicator of immunosuppression in sepsis. Research has demonstrated a reduction in CD4^+^ T lymphocytes, CD8^+^ T lymphocytes, and B lymphocytes in sepsis patients, correlating with their prognostic outcomes (Huang et al., 2024). Persistent lymphopenia is often indicative of poorer clinical outcomes and elevated mortality rates (Yang et al., 2024; Jing et al., 2024). Secondly, the expression level of mHLA-DR is recognized as a significant marker of immunosuppression in sepsis. Empirical evidence suggests that mHLA-DR expression on peripheral blood mononuclear cells below 15,000 antibodies/cell is indicative of immune insufficiency, while levels below 8,000 antibodies/cell denote severe immune paralysis (Joshi et al., 2023). The reduction in mHLA-DR expression is associated with suppressed immune function and is inversely correlated with sepsis severity and mortality (Joshi et al., 2023; Cui et al., 2025). Furthermore, the elevation of serum soluble PD-L1 (sPD-L1) levels is associated with the immunosuppressive state observed in sepsis patients. As an immune checkpoint molecule, increased sPD-L1 levels may indicate an inhibited immune response. Research has demonstrated a strong correlation between sPD-L1 levels and both the severity and prognosis of sepsis, suggesting its potential utility as a biomarker for assessing the immune status of sepsis patients (Zeng et al., 2023; Sun S. et al., 2021). Consequently, persistent lymphopenia, reduced expression of mHLA-DR, and elevated sPD-L1 levels serve as critical biomarkers of immunosuppression in sepsis. These markers not only aid in assessing the immune status of sepsis patients but also provide valuable insights for clinical decision-making, thereby potentially improving patient outcomes.

The application of immunotherapy, including IL-7 and immune checkpoint inhibitors (ICIs), in the management of sepsis (Francois et al., 2018; Hotchkiss et al., 2019a; Hotchkiss et al., 2019b).

Recently, the utilization of immunotherapy in the management of sepsis has attracted considerable scholarly interest. Sepsis is characterized as a systemic inflammatory response syndrome induced by infection. Conventional treatment modalities predominantly depend on antibiotics and supportive care. However, with an enhanced understanding of the immunopathophysiology of sepsis, immunotherapy has emerged as a promising therapeutic approach. IL-7, a critical cytokine, has been shown to restore T cell function in sepsis patients, augmenting both the proliferation and survival of T cells, thereby enhancing the body’s immune response (Ariail et al., 2025; Ahn et al., 2023). Furthermore, IL-7’s role in sepsis extends to the regulation of immune metabolism. Research indicates that IL-7 can ameliorate immune metabolic dysfunction in T lymphocytes of sepsis patients, promoting their proliferation and function through the activation of the mTOR pathway (Venet et al., 2017). Additionally, ICIs, such as nivolumab, have the capacity to block the PD-1/PD-L1 signaling pathway and restore T cell function, indicating potential for improving immune function in animal models of sepsis (McBride et al., 2020). A multi-center, open-label phase 1/2 study demonstrated that a single administration of 960 mg nivolumab was generally well tolerated in patients experiencing sepsis-induced immunosuppression. This treatment maintained blood concentration levels while progressively increasing the absolute lymphocyte count and the expression of the mHLA-DR subtype (Watanabe et al., 2020). In addition to IL-7 and ICIs, the use of intravenous immunoglobulin (IVIg) in patients with severe infections has been investigated. Although current evidence is insufficient to endorse its routine application in sepsis and septic shock, IVIg may offer benefits in specific contexts, such as streptococcal toxic shock syndrome (Aubron et al., 2019). Beyond pharmacological immunomodulation, extracorporeal blood purification techniques, including hemoadsorption, are being explored as adjunctive therapies to mitigate the dysregulated inflammatory response in sepsis, to restore immune homeostasis (Brendolan et al., 2024).

The literature co-citation analysis presented in Table 4 serves as a foundational tool for investigating research hotspots within this domain. Boomer et al. (2011) conducted an exploration into the characteristics and mechanisms underlying immunosuppression in sepsis patients, identifying a marked reduction in cytokine secretion (including TNF and IFN-γ) and a substantial depletion of immune effector cells such as CD4^+^ and CD8^+^ T cells. This immune dysfunction is potentially driven by a series of interconnected molecular cascade reactions. For instance, T cell depletion may be facilitated through the activation of both exogenous (Fas/FasL) and endogenous (mitochondrial) apoptosis pathways (Islam et al., 2024; Cao et al., 2019). Subsequent research has elucidated that Mfn2-mediated autophagy can expedite the apoptosis of CD4^+^ T cells, thereby exacerbating immunosuppression (Ying et al., 2017).

The prominent co-citation rankings of MDSCs (Mathias et al., 2017; Uhel et al., 2017) and the PD-1/PD-L1 signaling pathway (Chang et al., 2014; Patera et al., 2016) underscore their critical roles in sepsis-induced immunosuppression. Mathias et al. (2017) and Uhel et al. (2017) provide a comprehensive analysis of the expansion of MDSCs and low-density neutrophils (LDNs), elucidating a mechanism of cellular inhibition. These cells exert their immunosuppressive effects through various mechanisms: they secrete immunosuppressive cytokines, upregulate the activity of arginase 1 (ARG1) to deplete L-arginine—a vital amino acid for T cell proliferation—and, importantly, express elevated levels of PD-L1. Additionally, Chang et al. (2014) and Patera et al. (2016) highlight the interaction between PD-L1 and PD-1 on T cells, which can lead to T cell depletion and apoptosis. The PD-1/PD-L1 axis serves not only as a biomarker but also as a functional mediator of immunosuppression, as *in vitro* studies have demonstrated that specific antibody blockade can reverse T cell dysfunction and diminish apoptosis. Furthermore, Francois et al. (2018) demonstrated that recombinant human interleukin-7 (CYT107) significantly enhances the population of CD4^+^ and CD8^+^ T cells in patients with sepsis. IL-7 therapy mitigates the apoptosis of these critical cell types by promoting T cell proliferation and survival, and it has been shown to restore their metabolic function through the activation of the mTOR signaling pathway (Venet et al., 2017).

In recent years, intestinal microflora has emerged as a focal point of research in the context of sepsis. Investigations in this domain underscore the significant role of intestinal microflora in the pathogenesis of sepsis, particularly in its capacity to regulate host immune responses and sustain intestinal barrier integrity. Evidence indicates that dysbiosis, or imbalance of intestinal microflora, is closely associated with both susceptibility to sepsis and clinical outcomes (Piccioni et al., 2024; Lindner et al., 2023). Furthermore, intestinal microflora exerts direct effects on critical targets of the immune response through its metabolites, including short-chain fatty acids (SCFAs), bile acids, and indoleacetic acid (Li et al., 2024). These metabolites are instrumental in modulating inflammatory responses and preserving intestinal barrier function (Xu, 2025; Zhang Z. et al., 2024; Miao et al., 2025). Additionally, dysbiosis can lead to intestinal barrier dysfunction, thereby exacerbating the severity of sepsis (Piccioni et al., 2024; A et al., 2020).

Therapeutic strategies targeting intestinal microflora have garnered increasing academic interest. These strategies encompass selective decontamination of the digestive tract (SDD), probiotics, prebiotics, synbiotics, metazoa, and fecal microbial transplantation (FMT) (Piccioni et al., 2024; Kullberg et al., 2021; Miao H. et al., 2024). Notably, FMT has demonstrated a protective role in reestablishing intestinal microflora equilibrium, particularly within sepsis models (Han et al., 2024). Nonetheless, further clinical validation of these treatments is required. Additionally, the intricate relationship between intestinal microflora and cell death mediated by inflammasomes has emerged as a significant research focus. Evidence suggests that intestinal microflora can modulate the inflammatory response by affecting the activation of NLRP3 inflammasomes, thereby offering a novel avenue for future investigations (Zhang H. et al., 2024). Concurrently, the causal relationship between intestinal microflora and septicemia has been further examined through Mendelian randomization analysis, which highlights the potential intermediary role of C-reactive protein (CRP) in this process (Zhang Z. et al., 2023).

In summary, the role of intestinal microflora in sepsis and its potential therapeutic applications offer a novel perspective and direction for research in this domain. Future investigations should aim to elucidate the precise composition of intestinal microflora and its optimal state in sepsis, to develop more precise and effective treatment strategies (Yang Z. et al., 2025).

### 4.3 The evolution of hotspots and emerging themes

The timeline viewer (Figure 6) provided a comprehensive overview of the research trajectory in the field of sepsis-related immunosuppression over the past decade. During the initial research phase from 2005 to 2011, scholars concentrated on elucidating the fundamental relationship between sepsis and immunosuppression. Keywords such as “immunosuppression,” “sepsis,” and “septic shock” prominently feature on the timeline, signifying the primary research focus on understanding these phenomena and their directly associated factors. Concurrently, the appearance of keywords like “mortality,” “inflammation,” “survival,” and “efficacy” underscores the researchers’ concern regarding the severe consequences of sepsis and its impact on patient prognosis. Furthermore, the presence of terms such as “cytokines,” “activation,” and “upregulation” indicates that early investigations aimed to decipher the mechanisms underlying immunosuppression by examining immune cell activation and the expression changes of related molecules. These foundational studies paved the way for subsequent exploration of immune mechanisms.

During the intermediate phase of research from 2012 to 2020, there was a discernible transition in focus from examining basic correlations to conducting an in-depth exploration of the immunosuppressive mechanisms associated with sepsis. This shift is evidenced by the increased prominence of keywords such as pathways, suppressor, blockade, molecular mechanisms, PD-1, and IL-7. Researchers have dedicated their efforts to elucidating the mechanisms underlying sepsis-related immunosuppression and developing therapeutic strategies aimed at modulating immune cell function and rectifying immunosuppression. Furthermore, the growing emphasis on “biomarkers” highlights the need for reliable markers for early diagnosis and disease assessment, aiming to improve diagnostic accuracy and treatment specificity. These emerging research focal points illustrate a clear evolutionary trend over time, reflecting the deepening and broadening of research endeavors during this intermediate stage.

During the recent research phase spanning from 2021 to 2025, investigations into sepsis-related immunosuppression have exhibited a dispersed nature. The breadth of keywords during this period is notably extensive, while their frequency of occurrence remains comparatively low, rendering it challenging to distinctly identify concentrated and representative research hotspots. This dispersion may suggest that the field is experiencing a bottleneck, prompting researchers to explore multiple perspectives and directions in an effort to discover new breakthroughs and focal points for future study.

Due to the limitations of keyword analysis in accurately capturing current research trends, this study employed a burst citation analysis to identify emerging topics within the field of sepsis-related immunosuppression. Through this method, we identified the top 50 burst citations (Figure 7) and analyzed the representative studies. Our analysis revealed two potential emerging themes.

The application of ICIs in patients with sepsis-related immunosuppression (Hotchkiss et al., 2019b).

ICIs have significantly advanced cancer treatment; however, their application in sepsis remains in the exploratory phase. In sepsis patients, the expression of immune checkpoint molecules is elevated, which contributes to immunosuppression by inhibiting T-cell function and the activity of other immune cells (Patera et al., 2016; McBride et al., 2020; Wu et al., 2023). Consequently, inhibitors targeting these immune checkpoints are considered a potential therapeutic strategy to enhance the body’s resistance to infection by alleviating immunosuppression (Rienzo et al., 2022). Nonetheless, several challenges associated with the use of ICIs in sepsis treatment must be acknowledged. Primarily, the immune status of sepsis patients is complex and variable, and ICIs may exacerbate immune overactivation in certain cases, resulting in adverse events (Xia et al., 2022). Additionally, the administration of ICIs may elevate the risk of secondary infections (Abers and Lionakis, 2020). Despite these challenges, the potential application of ICIs in sepsis warrants further investigation. Future research should focus on identifying the subpopulation of sepsis patients most suitable for ICI treatment and determining the optimal timing and regimen for therapy (Nakamori et al., 2020).

The comprehension of the PD-1/PD-L1 pathway is crucial for advancing the clinical translation of ICIs. Consequently, future research must rigorously demonstrate the efficacy of ICIs in this patient population through extensive experimental studies. As indicated by our analysis of the molecular mechanisms, it is imperative to identify specific patient subgroups that are most likely to benefit from ICIs. This can be achieved by developing robust biomarker panels, such as the combination of mHLA-DR expression, PD-L1 levels, and lymphocyte counts, which can be dynamically stratified based on patients’ immune status. Furthermore, the optimal timing, dosage, and potential combinations of ICIs with other immune adjuvants, such as IL-7, require thorough investigation in meticulously designed clinical trials to maximize therapeutic efficacy while minimizing the risk of immune-related adverse events.

Exploring the regulatory mechanism of immune cell apoptosis induced by sepsis (Cao et al., 2019).

In the pathophysiological process of sepsis, the apoptosis of immune cells is considered to be one of the main causes of immunosuppression (Cao et al., 2019). This apoptotic process encompasses not only adaptive immune cells, such as T cells and B cells, but also innate immune cells, including macrophages and dendritic cells (Nedeva, 2021). Immune cell apoptosis induced by sepsis involves complex molecular mechanisms and signal pathways. Firstly, sepsis-induced apoptosis of immune cells can be mediated through endoplasmic reticulum stress and the mitochondrial pathway. Research indicates that endoplasmic reticulum stress significantly contributes to CD4^+^ T cell apoptosis and dysfunction; thus, its inhibition can mitigate CD4^+^ T cell apoptosis and maintain their functional capacity (Lu et al., 2022). Furthermore, the mitochondrial-mediated apoptosis pathway is also activated during sepsis. Overexpression of thioredoxin-1 (Trx-1) has been shown to decrease the apoptosis of splenic cells by inhibiting the mitochondrial-mediated apoptosis pathway, thereby offering protective effects (Chen et al., 2017). Secondly, sepsis influences the apoptosis of immune cells through the modulation of immune checkpoint molecules. For instance, the expression of the immune checkpoint molecule Tim-3 is upregulated in natural killer T (NKT) cells, thereby promoting their apoptosis and correlating with poor prognosis in sepsis patients (Wu et al., 2023). Moreover, the PD-1/PD-L1 axis serves as a critical regulator of immunosuppression in sepsis, exacerbating immunosuppression by inhibiting immune cell activation and promoting apoptosis (Văsiesiu et al., 2025). Thirdly, sepsis modulates the survival of immune cells by influencing the equilibrium between autophagy and apoptosis. Research has demonstrated that Pink1/Parkin-mediated mitochondrial autophagy is activated during sepsis, which exerts an anti-apoptotic effect on dendritic cells, thereby influencing immune function (Zhang et al., 2022). Furthermore, mitochondrial fusion protein 2 (Mfn2) facilitates the apoptosis of CD4^+^ T cells by inhibiting autophagy, suggesting that autophagy deficiency plays a crucial role in sepsis-induced apoptosis of immune cells (Ying et al., 2017). Understanding the mechanisms underlying sepsis-induced immune cell apoptosis and developing intervention strategies is of paramount importance for the advancement of effective therapeutic approaches.

The citation burst analysis (Figure 7) shows that the application of ICIs and the exploration of immune cell apoptosis regulation mechanisms are emerging research topics in this research field. Within the context of sepsis research, the PD-1/PD-L1 pathway is recognized as a critical factor contributing to immunosuppression. PD-L1, a co-inhibitory molecule, is predominantly expressed on the surface of immune cell membranes. By interacting with PD-1, it suppresses T cell proliferation and cytokine secretion, thereby modulating the adaptive immune response. Nevertheless, the function of PD-L1 in sepsis extends beyond immunosuppression and may include a pro-inflammatory role. For instance, research has demonstrated that PD-L1 can influence chemokine production *via* the TLR4/TRAF6 signaling axis under septic conditions, consequently affecting immune cell recruitment and the inflammatory response (Yuan et al., 2026). Therapeutic strategies targeting the PD-1/PD-L1 pathway exhibit promising potential in the treatment of sepsis. For example, PD-L1 expression is associated with decreased neutrophil function, and inhibiting PD-1 or PD-L1 can restore neutrophil function and enhance the immune status of sepsis patients (Liu et al., 2024). Moreover, the apoptosis of immune cells is a critical factor in the immunosuppression associated with sepsis. The mTOR pathway is pivotal in modulating the apoptosis of CD4^+^ T cells triggered by endoplasmic reticulum stress (ERS). Inhibition of autophagy by the mTOR pathway can exacerbate ERS-induced apoptosis, whereas the absence of mTOR can mitigate this apoptosis by reinstating autophagy (Lei et al., 2026). Furthermore, PIK3C3, as a key regulator of autophagy, significantly influences immune cell function. It modulates the activation, proliferation, survival, and apoptosis of immune cells through the regulation of autophagy, thereby exerting an immunomodulatory effect in the pathophysiological context of sepsis (Wei et al., 2025). Consequently, targeting mTOR or PIK3C3 may represent a promising therapeutic strategy to restore immune homeostasis and enhance the clinical outcomes in sepsis.

### 4.4 The future research direction and prospect

Sepsis-related immunosuppression represents a critical phase in the progression of sepsis, necessitating interdisciplinary research that encompasses immunology, inflammatory responses, cell signaling pathways, and clinical treatment strategies. Although recent years have witnessed advancements in elucidating mechanisms and developing treatment protocols, numerous enigmas and unmet clinical needs persist. Current research predominantly investigates the mechanisms underlying immune cell dysfunction, the role of immune checkpoint molecules (such as the PD-1/PD-L1 pathway), the function of MDSCs, apoptosis, metabolic reprogramming, and the preliminary exploration of immunotherapeutic approaches, including ICIs. Nonetheless, the majority of these studies remain in the basic research phase or early clinical trials and have yet to be fully integrated into standardized clinical treatment protocols.

Future research may focus on several key areas. Firstly, there is a need to deepen the understanding of the molecular mechanisms underlying immunosuppression. This can be achieved by analyzing the functional state and molecular characteristics of immune cell subsets at various stages of sepsis using single-cell sequencing technology. Integrating data from transcriptomics, proteomics, and metabolomics will facilitate the construction of a comprehensive map of immunosuppression. Secondly, the development of novel biomarkers and diagnostic tools is crucial. This involves creating biomarker combinations that can dynamically reflect the state of immunosuppression by integrating multi-dimensional data, including cell surface markers, cytokines, and metabolites. Thirdly, optimizing immunotherapy strategies is essential. This includes identifying patient groups suitable for immunotherapy through biomarker screening, optimizing the timing and dosage of treatment, and enhancing the efficacy and safety of therapeutic interventions. Lastly, conducting long-term follow-up studies on sepsis survivors will allow for the assessment of immune function recovery trajectories and their impact on reinfection, chronic diseases, and quality of life.

To promote research progress, it is necessary to adopt advanced research methods and technologies. For instance, artificial intelligence and big data analytics can leverage machine learning algorithms to analyze multi-source data—including clinical, laboratory, and imaging data—pertinent to sepsis. This approach facilitates the construction of predictive models aimed at early warning and precise intervention in cases of immunosuppression. Additionally, organ-like and humanized models offer enhanced simulation of the pathophysiological processes associated with human sepsis, thereby expediting the validation and refinement of novel therapeutic strategies. Furthermore, innovative clinical trial designs, such as adaptive clinical trial frameworks and real-world evidence studies, can enhance the efficiency and feasibility of immunotherapy trials, accelerating the translation of research findings into practice. Through interdisciplinary collaboration and fusion of innovative approaches, it is expected to advance research in the field of sepsis-related immunosuppression and ultimately improve patient clinical outcomes.

### 4.5 Limitations

This study is subject to two intrinsic limitations associated with bibliometric methods. Firstly, the primary data source utilized is the WoSCC database, which is widely regarded as the gold standard for bibliometric research due to its extensive citation data and standardized metadata. Nevertheless, some studies published in other databases, non-English journals, or regional databases have not been included in the analysis, which may cause some bias to the research results. To further assess the robustness of our results, we employed the PubMed database for validation, given its status as an authoritative source in the field of biomedical science. The data obtained from these different sources yielded similar research outcomes, thereby demonstrating the reliability of our findings. Secondly, our analysis prioritizes quantitative metrics intrinsic to the literature itself, such as the number of publications, citation frequency, and co-citation frequency, rather than focusing on journal-specific indicators like impact factors. While the impact factor serves as a metric for assessing the average influence of a journal, it does not inherently reflect the quality or impact of individual articles. Nonetheless, incorporating impact factors could offer an additional dimension for exploration within this field. Future research could enhance our analysis by integrating journal impact factors, thereby offering a more nuanced understanding of the research landscape.

## 5 Conclusion

This study systematically elucidates the research landscape of sepsis-related immunosuppression spanning from 2005 to 2025. The global research trajectory is characterized by phases of “initiation, rapid growth, and plateau,” with the United States, China, and France emerging as pivotal contributors, notably with significant input from institutions such as INSERM in France. The research predominantly concentrates on five key areas: the mechanisms underlying immune cell dysfunction, regulation of the PD-1/PD-L1 pathway, inhibition of myeloid cells, assessment of immune markers, and the investigation of immunotherapeutic approaches. Recent research trends have shifted towards the clinical translation of ICIs and the regulation of immune cell apoptosis. However, the field has reached a plateau and necessitates breakthroughs in understanding mechanisms and developing treatment strategies. Overall, sepsis-related immunosuppression remains a dynamic and evolving area of research, with its findings holding substantial implications for enhancing the treatment and prognosis of sepsis patients. Future research needs to deepen the understanding of the immunosuppression mechanism and explore new treatment strategies to provide more effective clinical intervention measures for sepsis patients.

## Data Availability

The original contributions presented in the study are included in the article/supplementary material, further inquiries can be directed to the corresponding author.
